# First record of the dotted grouper *Epinephelusepistictus* (Temminck & Schlegel, 1843) (Perciformes, Serranidae) in Malaysia

**DOI:** 10.3897/zookeys.861.34043

**Published:** 2019-07-08

**Authors:** Jianguo Du, Kar-Hoe Loh, Amy Yee-Hui Then, Xinqing Zheng, Teguh Peristiwady, Mohammed Rizman-Idid, Man Alias

**Affiliations:** 1 Third Institute of Oceanography, Ministry of Natural Resources, Xiamen 361005, China; 2 Institute of Ocean and Earth Sciences, University of Malaya, Kuala Lumpur 50603, Malaysia; 3 Institute of Biological Sciences, Faculty of Science, University of Malaya, Kuala Lumpur 50603, Malaysia; 4 Bitung Marine Life Conservation, Research Center for Oceanography, Indonesian Institute of Sciences, Bitung 97255, North Sulawesi, Indonesia; 5 Planning and Development Division, Department of Fisheries Malaysia, Putrajaya 62628, Malaysia

**Keywords:** DNA barcoding, new record, otolith aspect ratio, phylogenetic and genetic diversity, taxonomy

## Abstract

Five specimens of *Epinephelusepistictus* (Temminck & Schlegel, 1843) were collected from a major landing site located on the west coast of Peninsula Malaysia during a fish faunal survey on 23 August 2017. The present study extends the distribution range of *E.epistictus* southwards from Andaman Sea to the Strait of Malacca. Species identification was confirmed by colour pattern and DNA barcoding (567 bp of cytochrome C oxidase I) of all *E.epistictus* specimens and nine closely related *Epinephelus* species. The interspecies genetic distance ranged from 0.002–0.245. This study also presents, for the first time for Malaysia, data on length-weight relationships and otolith measurements. It contributes to a better understanding of taxonomy, and phylogenetic and genetic diversity of *E.epistictus*.

## Introduction

Groupers (subfamily Epinephelinae of the family Serranidae) are a commercially valuable taxa globally and in Malaysia, in particular (Craig et al. 2011; Bray 2018; Froese and Pauly 2018). To date, a total of approximately 65 species in 15 genera have been reported in Malaysia (Department of Fisheries Malaysia 2009; Chong et al. 2010; Matsunuma et al. 2011; Ambak et al. 2012; Yusri et al. 2015).

The dotted grouper *Epinephelusepistictus* (Temminck & Schlegel, 1843) is in the Least Concern (LC) category (Leung and Sadovy 2018). It is a demersal species, which inhabits the continental shelf over soft or rocky bottom at a considerable depth (71–800 m); however little else is known about this species (Paxton et al. 1989; Heemstra and Randall 1993; Goldshmidt et al. 1996; Sommer et al. 1996; To and Pollard 2008). This is a widely distributed species in the Indo-West Pacific, occurring off South Africa, in the Red Sea (Randall and Adam 1983), off Iraq (Mustafa et al. 2011), Oman (Krupp et al. 2000), on the west coast of India, Korea, Japan, in the South China Sea (Randall and Lim 2000), off Taiwan, Hong Kong, Indonesia (Limmon et al. 2017; Mous and Pet 2018), Papua New Guinea (Froese and Pauly 2018), and northern Australia (Bray 2018; Dianne 2019). It can be identified by a pale brown body with irregular rows of small dark spots on the back and sides of the body. Some specimens have a broad dark band from the eye to the gill cover, and two narrower bands running diagonally across the cheek (Bray 2018).

*Epinephelusepistictus* is a medium-sized grouper, with a maximum of 80 cm total length, and may be misidentified as *Epinephelusmagniscuttis* Postel, Fourmanoir & Guézé, 1963 or *Epinephelusheniochus* Fowler, 1904 (To and Pollard 2008; Froese and Pauly 2018; Leung and Sadovy 2018). *E.epistictus* and *E.heniochus* differ from *E.magniscuttis* by having fewer and smaller dark spots on head and body and dark spots arranged in three longitudinal rows on body of juveniles (Heemstra and Randall 1993). However, *E.heniochus* and *E.epistictus* share the following characters: distinctly enlarged serrae at the corner of the preopercle, 14 or 15 dorsal-fin rays, interspinous dorsal-fin membranes distinctly incised, midlateral part of lower jaw with two rows of teeth, similar morphometric features and colour pattern (Heemstra and Randall 1993). These similarities might have contributed to *E.epistictus* misidentifications in the past. Though a number of barcoding (CO1) studies of groupers from Malaysia have been conducted (Chu et al. 2011; Nurnadia et al. 2016; Rahim et al. 2016), the species occurrence remained undetected. A recent ichthyofaunal survey found specimens of *E.epistictus* in a commonly surveyed major landing site on the west coast of Peninsula Malaysia and the present study reports on size and genetic data that confirm its identification as well as some aspects of its biology and phylogeny.

## Materials and methods

**Figure 1.**
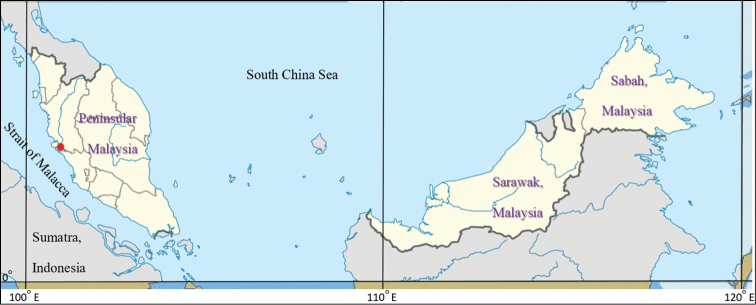
The map of Hutan Melintang (red dot) showing the location of the landing site.

Five specimens of *E.epistictus* were collected from a major fish landing site in Hutan Melintang, northeastern Peninsula Malaysia during a fish faunal survey on 23 August 2017 (Fig. 1). These specimens were caught using trawl nets operating in the Straits of Malacca. The tissue samples were preserved in 95% ethanol solution and deposited in the Institute of Ocean and Earth Sciences (IOES), University of Malaya (UM), Kuala Lumpur. Preliminary species identification was made based on the morphology of the whole fish specimens using the species identification keys and diagnostic features reported in Heemstra and Randall (1993). Morphological measurements taken included total length (TL, mm), standard length (SL, mm), and total weight (Wt, g). Otoliths were extracted and various measurements were made, namely the sagitta otolith length (O_L_, mm), the longest distance between the most anterior and posterior points, otolith width (O_w_, mm), the longest distance between the ventral and dorsal edges, and the weight of the sagittal (O_wt_, g). Otolith aspect ratio (O_AS_) was calculated by dividing O_L_ by O_W_ for the left otolith (Table 1).

**Table 1.** Morphometric measurements for *Epinephelusepistictus*.

**Measurements**	**DOS339**	**DOS340**	**DOS341**	**DOS342**	**DOS370**
TL (mm)	180	164	158	150	145
SL (mm)	154	135	130	122	127
Wt (g)	70.75	59.25	49.30	41.55	36.44
Otolith morphometrics					
Left					
O_L_ (mm)	8.17	8.57	7.43	7.59	8.12
O_W_ (mm)	3.98	3.84	3.78	3.81	3.69
O_Wt_ (g)	0.0284	0.0292	0.0238	0.0241	0.0228
Right					
O_L_ (mm)	7.09+	8.42	7.36	7.57	8.07
O_W_ (mm)	3.87+	3.85	3.64	3.78	3.61
O_Wt_ (g)	0.0272+	0.0291	0.0235	0.0238	0.0225

Note: + broken; total lenght (TL), standard length (SL), total weight (Wt); otolith length (O_L_), Otolith width (O_W_), weight of the otolith (O_Wt_).

Molecular DNA sequencing was used to confirm the species identification. Total DNA extractions were performed on the collected tissue samples using the G-spin^TM^ Total DNA Extraction Kit (iNtRON Biotechnology, Inc., Korea) following the manufacturer’s instructions. The primers, including combinations of the forward (FishF1 or FishF2) and the reverse (FishR1 or FishR2) primer pairs, followed Ward et al. (2005). A 20 μl PCR reaction mixture was prepared in a 1.5 ml tube containing 13.25 μl double distilled water (ddH_2_O), 2 μl 10x *i*-Taq plus PCR buffer, 1 μl of deoxynucleotide triphosphate (dNTP), 1 μl of each primer used, 0.25 μl *i*-Taq plus DNA polymerase and 1.5 μl of total genomic DNA. The Eppendorf thermal cycler was used to run the following thermal cycle profile: initial denaturation at 94 °C for 5 minutes; 35–40 cycles of denaturation at 94 °C for 30s, annealing at 44–50 °C for 30s, extension at 72 °C for 1 minute; followed by a final extension at 72 °C for 5 minutes. The PCR products were stained with loading dye, and loaded on to the wells of 1.0% agarose gel before conducting gel electrophoresis. Successfully amplified PCR products were sent to 1^st^ BASE Laboratories (Malaysia) with the same primers used for PCR reactions for sequencing.

For systematic relationships with congeners, the raw sequences were first assembled and edited via ChromasPro ver 1.42 (Technelysium Pty Ltd), subsequently aligned using Clustal X v. 2.0.8 (Larkin et al. 2007) and then manually adjusted with Bioedit v. 7.0.9.0 (Hall 1999). The COI gene of the five *E.epistictus* specimens, and nine closely related species (*Epinephelusbleekeri* (Vaillant, 1878), *Epinephelusfuscoguttatus* (Forsskål, 1775), *Epinepheluslatifasciatus* (Temminck & Schlegel, 1843), *Epinephelusquoyanus* (Valenciennes, 1830), *Epinephelusareolatus* (Forsskål, 1775), *Epinepheluscoioides* (Hamilton, 1822), *Epinepheluserythrurus* (Valenciennes, 1828), *E.heniochus*, and *Epinephelussexfasciatus* (Valenciennes, 1828)) sampled from Malaysian waters were also sequenced. Other sequences available in GenBank of *E.epistictus*, *E.bleekeri*, *E.fuscoguttatus*, *E.heniochus*, *E.latifasciatus* and *E.quoyanus* were also added to the analysis using the slender grouper, *Anyperodonleucogrammicus* (Valenciennes, 1828) (GQ131336) as outgroup (Table 2). The maximum likelihood (ML) tree was reconstructed based on the best evolutionary model, namely the General Time Reversible model (GTR) with the Gamma distributed (G) distance and invariable sites (I), which was selected using the lowest bias-corrected Akaike Information Criterion (AICc) value in model test, with 1000 replications for bootstrap analysis. Both tree construction and model test were completed in MEGA (Molecular Evolution Genetic Analysis) version 7.0 (Kumar et al. 2016). Similarly, a neighbor joining (NJ) tree was constructed based on the pairwise genetic distance using the Kimura 2-parameter (K2P) model with 1000 bootstrap resampling. Genetic distances of the sequences were calculated with the K2P model (Kimura 1980) using MEGA 7.0.

**Table 2.** Accession numbers of sequences used in the analysis and voucher catalogue numbers.

**Species**	**Location**	**Accession number**	**Reference**
* Epinephelus areolatus *	Malaysia (PK 011)	JN208570	This study (Rizman-Idid et al.)
	Malaysia (PK 017)	JN208571	This study (Rizman-Idid et al.)
* E. bleekeri *	USA	JN021297	Shen and Ishida (2016)
	Philippines	KU668653	Cabana (2017)
	Malaysia (DOS 361)	MK118153	This study
* E. coioides *	Malaysia (LK 021)	JN208587	This study (Rizman-Idid et al.)
	Malaysia (LK 033)	JN208589	This study (Rizman-Idid et al.)
* E. epistictus *	Saudi Arabia	KU499627	Rabaoui et al. (2016)
	India	KM226255	Vineesh et al. (2014)
	Malaysia (DOS 339)	KM118148	This study
	Malaysia (DOS 340)	KM118149	This study
	Malaysia (DOS 341)	KM118150	This study
	Malaysia (DOS 342)	MK118151	This study
	Malaysia (DOS 370)	MK118152	This study
* E. erythrurus *	Malaysia (LK 039)	JN208608	This study (Rizman-Idid et al.)
	Malaysia (LK 073)	JN208609	This study (Rizman-Idid et al.)
* E. fuscoguttatus *	Malaysia (PG 016)	JN208615	This study (Rizman-Idid et al.)
	Andaman, India	JX674997	Sachithananda et al. (2012)
* E. heniochus *	China	MF185518	Qu et al. (2018)
	Malaysia	KY371468	Hou et al. (2017)
	Malaysia (DOS 343)	MK118155	This study
	Malaysia (DOS 344)	MK118156	This study
* E. latifasciatus *	China	KC480177	Lai et al. (2013)
	China	MF185521	Qu et al. (2018)
	Malaysia (DOS 369)	MK118154	This study
* E. quoyanus *	Malaysia (LK 058)	JN208619	This study (Rizman-Idid et al.)
	China	MF185570	Qu et al. (2018)
* E. sexfasciatus *	Malaysia (LK 035)	JN208565	This study (Rizman-Idid et al.)
	Malaysia (LK 053)	JN208566	This study (Rizman-Idid et al.)
* Anyperodon leucogrammicus *	China	GQ131336	Lin et al. (2016)

## Results

**Figure 2.**
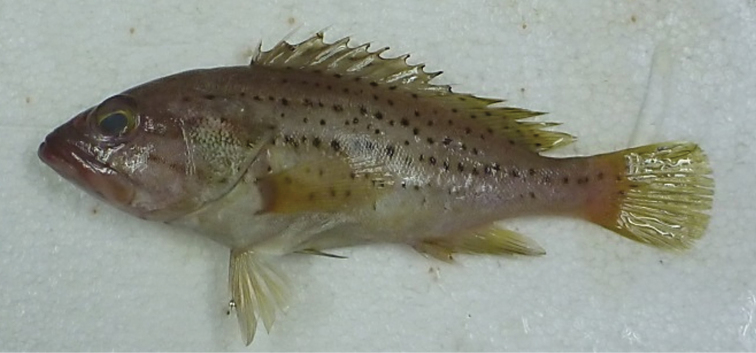
*Epinephelusepistictus* (DOS339), 180 mm TL, 70.75 g.

**Figure 3.**
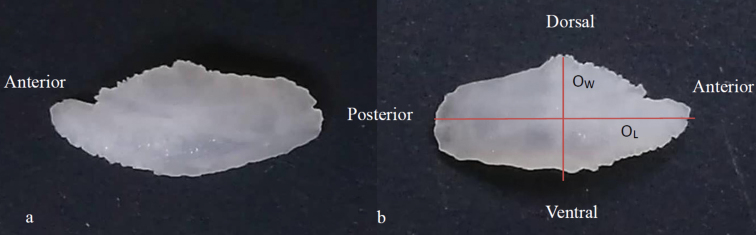
Sagittae of *Epinephelusepistictus* DOS370 **a** right otolith **b** left otolith, the positioning of otolith morphometrics measured (in mm), O_L_ (otolith length) the longest distance between the most anterior and posterior points; O_w_ (Otolith width) the longest distance between the ventral and dorsal edges.

The colour pattern of the Malaysian specimens identified as *E.epistictus* was similar to the species’ description in Bray (2018) (Fig. 2). The five specimens were 122–154 mm SL (145–180 mm TL) and 36.44–70.75 g total weight. Estimates of the length-weight parameters were -2.0542 for log *a* (95% CI = -2.1378, -2.0353). The length-weight relationship based on total length and total weight showed positive allometric growth (*b* = 3.1245) and the coefficient of determination (r^2^) of the regression was 0.9725. Otolith measurements were presented using mean (standard deviation): O_L_ 7.98 (0.46) mm; O_w_ 3.82 (0.11) mm; and O_W_t 0.0257 (0.0029) g (Table 1). The otolith aspect ratio (O_AS_) averaged 2.09 (0.12) for the left otolith (Fig. 3).

The 19 grouper specimens were classified as ten species based on external morphology, which was consistent with the species names in both the GenBank BLAST and BOLD-IDS. DNA sequences from the above said ten species were submitted to GenBank (PubMed) and their accession number were given in Table 2. There was a total of 181/567 bp of variable sites and 174/567 bp of parsimony-informative sites after aligning the sequences. The average base composition obtained was 25.0% A bases, 27.7% of C bases, 16.4% of G bases and 30.9% of T bases.

**Figure 4.**
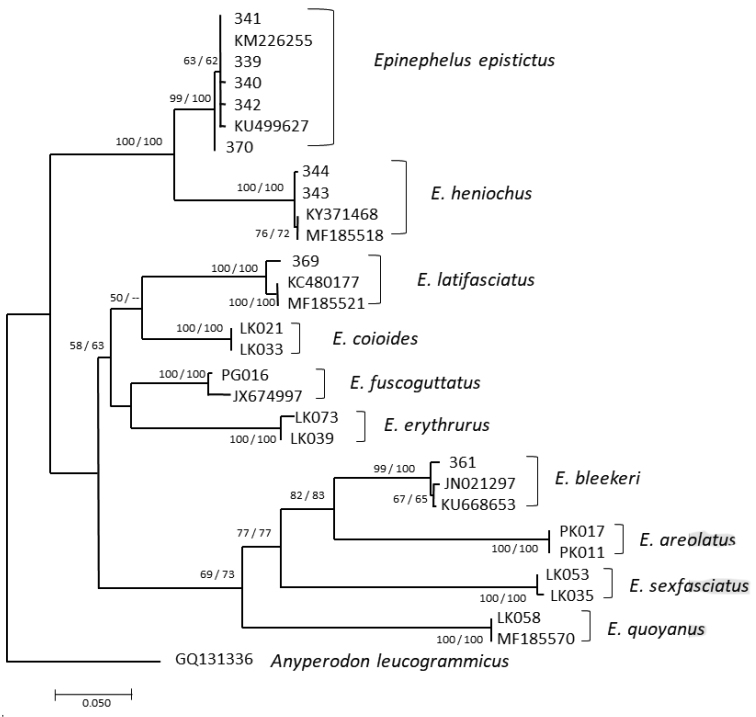
Phylogenetic inferred based on COI gene sequences (567 bp) for the ten *Epinephelus* species. The bootstrap values higher than 50% are shown at the branching points, methods ML/ NJ.

Both ML and NJ trees showed two major groups: one group containing of *E.epistictus*, *E.heniochus* and the other comprising other *Epinephelus* species. All species are monophyletic with bootstrap values range of 63–100%. All five *E.epistictus* samples were clustered together with the *E.epistictus* reference sequences from GenBank (KM226255, KU499627) with high bootstrap values (63–99% in ML tree, 62–100% in NJ tree) (Fig. 4).

Genetic distances between grouper species based on the 567 bp COI consensus sequences and the respective reference sequences with the K2P model were given in Table 3. The intraspecific nucleotide distances for *E.epistictus* were low, ranging from 0.000–0.005. The interspecific differences between other grouper species of the genus *Epinephelus* ranged from 0.002–0.245. The largest difference was found between *E.heniochus* and *E.sexfasciatus* (0.245).

**Table 3.** Pairwise comparisons of genetic distances (*d*) within all the grouper samples.

	1	2	3	4	5	6	7	8	9	10	11	12	13	14	15	16	17	18	19	20	21	22	23	24	25	26	27	28	29
1																													
2	0.002																												
3	0.000	0.002																											
4	0.002	0.004	0.002																										
5	0.004	0.005	0.004	0.005																									
6	0.002	0.004	0.002	0.004	0.005																								
7	0.000	0.002	0.000	0.002	0.004	0.002																							
8	0.082	0.084	0.082	0.084	0.082	0.082	0.082																						
9	0.084	0.087	0.084	0.087	0.084	0.084	0.084	0.002																					
10	0.084	0.087	0.084	0.087	0.084	0.084	0.084	0.002	0.004																				
11	0.084	0.087	0.084	0.087	0.084	0.084	0.084	0.002	0.004	0.000																			
12	0.174	0.174	0.174	0.177	0.168	0.174	0.174	0.189	0.186	0.192	0.192																		
13	0.180	0.180	0.180	0.183	0.174	0.180	0.180	0.186	0.189	0.189	0.189	0.016																	
14	0.180	0.180	0.180	0.183	0.174	0.180	0.180	0.186	0.189	0.189	0.189	0.016	0.000																
15	0.204	0.200	0.204	0.207	0.197	0.204	0.204	0.194	0.198	0.198	0.198	0.190	0.187	0.187															
16	0.204	0.200	0.204	0.207	0.197	0.204	0.204	0.198	0.201	0.201	0.201	0.181	0.178	0.178	0.009														
17	0.204	0.200	0.204	0.207	0.197	0.204	0.204	0.198	0.201	0.201	0.201	0.184	0.181	0.181	0.011	0.005													
18	0.187	0.190	0.187	0.187	0.187	0.190	0.187	0.209	0.212	0.212	0.212	0.200	0.200	0.200	0.130	0.132	0.130												
19	0.187	0.190	0.187	0.187	0.187	0.190	0.187	0.209	0.212	0.212	0.212	0.200	0.200	0.200	0.130	0.132	0.130	0.000											
20	0.138	0.138	0.138	0.136	0.138	0.138	0.138	0.158	0.155	0.161	0.161	0.117	0.109	0.109	0.180	0.174	0.174	0.200	0.200										
21	0.138	0.138	0.138	0.136	0.138	0.138	0.138	0.158	0.155	0.161	0.161	0.117	0.109	0.109	0.180	0.174	0.174	0.200	0.200	0.000									
22	0.172	0.172	0.172	0.175	0.178	0.172	0.172	0.188	0.185	0.191	0.191	0.135	0.138	0.138	0.175	0.166	0.166	0.180	0.180	0.142	0.142								
23	0.169	0.169	0.169	0.172	0.169	0.169	0.169	0.185	0.181	0.188	0.188	0.133	0.135	0.135	0.169	0.160	0.160	0.177	0.177	0.139	0.139	0.009							
24	0.144	0.144	0.144	0.146	0.138	0.144	0.144	0.167	0.170	0.170	0.170	0.127	0.125	0.125	0.145	0.142	0.142	0.198	0.198	0.117	0.117	0.123	0.115						
25	0.143	0.143	0.143	0.146	0.138	0.143	0.143	0.172	0.175	0.175	0.175	0.134	0.131	0.131	0.161	0.158	0.158	0.197	0.197	0.124	0.124	0.122	0.119	0.014					
26	0.204	0.207	0.204	0.207	0.197	0.200	0.204	0.242	0.238	0.245	0.245	0.192	0.199	0.199	0.167	0.183	0.179	0.174	0.174	0.190	0.190	0.188	0.175	0.178	0.192				
27	0.200	0.204	0.200	0.204	0.194	0.197	0.200	0.238	0.234	0.242	0.242	0.196	0.196	0.196	0.164	0.179	0.176	0.171	0.171	0.187	0.187	0.184	0.172	0.175	0.189	0.002			
28	0.185	0.189	0.185	0.182	0.185	0.185	0.185	0.188	0.191	0.191	0.191	0.242	0.232	0.232	0.173	0.167	0.167	0.199	0.199	0.194	0.194	0.190	0.190	0.183	0.194	0.203	0.200		
29	0.185	0.189	0.185	0.182	0.185	0.185	0.185	0.188	0.191	0.191	0.191	0.242	0.232	0.232	0.173	0.167	0.167	0.199	0.199	0.194	0.194	0.190	0.190	0.183	0.194	0.203	0.200	0.000	
30	0.156	0.156	0.156	0.153	0.156	0.156	0.156	0.182	0.179	0.185	0.185	0.160	0.181	0.181	0.188	0.188	0.188	0.213	0.213	0.155	0.155	0.200	0.190	0.140	0.156	0.198	0.194	0.188	0.188

Genetic distances were calculated to Kimura 2-parameter (K2P) model. 1= DOS339, 2= DOS340, 3= DOS341, 4= DOS342, 5= DOS370, 6= KU499627, 7= KM226255, 8= DOS343, 9= DOS344, 10= MF185518, 11= KY371468, 12= DOS369, 13= MF185521, 14= KC480177, 15= DOS361, 16= KU668653, 17= JN021297, 18= PK011, 19=PK017, 20=LK033, 21=LK021, 22=LK073, 23=LK039, 24=PG016, 25= JX674997, 26=LK035, 27=LK053, 28=LK058, 29=MF185570, 30=GQ131336.

## Discussion

Our study confirmed a new record of *E.epistictus* in the waters of Malaysia (in the Strait of Malacca). Although the strait is one of the busiest shipping channels in the world, our discovery of this commercially important grouper species suggests that much work remains to be done with documenting the local fish diversity. While the strait itself is considerably shallow in the south (close to Singapore) with average minimum depth of 25 m, the northern part of the Strait connecting to the Andaman Sea is up to 200 m deep; this depth profile is consistent with the depth range at which the species reportedly occurs. An undergraduate thesis reported the use of this species at an aquaculture farm in the Sabahan Borneo (Chen 2015), however, examination of the photos included in the thesis revealed that the species was erroneously identified as *E.epistictus*. A morphometric comparison and characters distinguishing *E.epistictus* from *E.bleekeri*, *E.heniochus* and *E.latifasciatus* were compared with existing literature (Sommer et al. 1996; Krupp et al. 2000; Froese and Pauly 2018) in Appendix 1.

Our study demonstrated evidence to support fine-scale monophyly for the subset of *Epinephelus* species examined in this region based on COI sequence data. Despite comparison of specimens from various distant locations, namely India, Saudi Arabia and Malaysia, the intraspecific nucleotide distances of *E.epistictus* was relatively low (0.000–0.005). The interspecific differences between ten grouper species examined ranged from 0.002 to 0.245, and 0.213 from the closest outgroup *Anyperodonleucogrammicus*. This species was included by some (Craig and Hastings 2007, Ma and Craig 2018) in the genus *Epinephelus* but others have kept it in the monotypic genus (Rhodes 2018) until further phylogenetic study is done. A recent study placed groupers in the family Epinephelidae sensu Smith and Craig (2007) and supports the idea of assigning both *E.epistictus* and *E.heniochus* to *Mycteroperca* (Ma and Craig 2018). At the species level, the status of *E.epistictus* has not been questioned.

The findings of this study contribute to better understanding on the taxonomy, biology, phylogenetic and genetic diversity of *E.epistictus*, which is important for sustainable management of the species in Malaysia.

## Appendix 1

**Table:** Morphometric comparison and characteristics distinguishing *Epinephelusepistictus* from *E. bleekeri*, *E.heniochus*and *E.latifasciatus*(References: Sommer et al. 1996, Krupp et al. 2000, Cites 2017, Froese and Pauly 2018).

	*** E. epistictus *** **(Temminck & Schlegel, 1843)**	*** E. bleekeri *** ** (Vaillant, 1878)**	*** E. heniochus *** **Fowler, 1904**	*** E. latifasciatus *** **(Temminck & Schlegel, 1843)**
Commen name	Dotted grouper	Duskytail grouper	Bridled grouper	Striped grouper
Type locality	Japan	Indonesia	Indonesia	Japan
Holotype	RMNH D88 (stuffed)	MNHN 0000-7401	ANSP 27558	Lectotype: RMNH D21 (stuffed)
Dorsal fin	XI	XI	XI	XI
Dorsal rays	14–15	16–18	14–15	12–14
Anal spins	III	III	III	III
Anal rays	8	8-9.	8	8
Pectoral rays	18	17–19	16–18	17–19
Gill rakers	8+16	25–28 (9–11+16–18)	7–9+14–16	8–11+15–18
Lateral line scales	60	49–53	54–60	56–65
Lateral scale series	108	99–104	80–100	91–106
**in SL**				
Head length	2.4	2.4–2.7	2.2–2.4	2.3–2.6
Body depth	3.4	3.0–3.5	2.7–3.2	2.9–3.4
Colour	Light brownish on flanks with very small dark brown spots on upper sides of body; dorsal fins, pectoral fins, anal fin, caudal-fin membranes yellow	Head and body brownish, reddish brown or purplish grey, covered (except ventrally) with numerous reddish orange, gold, or yellow spots; dorsal fin and upper third of caudal fin with spots like those on body; lower two-thirds of caudal fin dusky	Head and body pale brown dorsally, shading to whitish or pale pink ventrally; faint dark brown stripe from eye to end of operculum; pectoral fins hyaline greyish yellow; margin of interspinous dorsal-fin membranes yellow	2 black-edged white longitudinal bands, upper band extending from above the eye to the anterior dorsal-fin rays, lower band from below the eye to the lower caudal-fin rays; black spots and streaks on dorsal and caudal fins; head and body of large adults, uniformly grey
Geographical distribution	Indo-West Pacific species	Indo-West Pacific species	West Pacific species	Indo-West Pacific species
Habitat	Rocky and trawlable bottoms	shallow rocky banks	mud or silty-sand bottom	Rocky, silty-sand and mud bottom
Depth	71–800 m	30–104 m	40–235 m	20–230 m
IUCN category	Least Concern (LC)	Near threatened (NT)	Least Concern (LC)	Least Concern (LC)
